# Effectiveness of Biphasic Calcium Sulfate Mineral Cement in the Treatment of Advanced Peri-Implantitis: A Case Report

**DOI:** 10.7759/cureus.107934

**Published:** 2026-04-29

**Authors:** Damian Dudek, Maciej Jagielak, Oliwia Warmusz, Edyta Reichmann-Warmusz

**Affiliations:** 1 Perioperative Dentistry, Faculty of Medicine, Ludwik Rydygier Collegium Medicum in Bydgoszcz, Nicolaus Copernicus University, Toruń, POL; 2 Oral and Maxillofacial Surgery, Private Practice, Raszyn, POL; 3 Histology, Silesian Nanomicroscopy Center, Silesia LabMed-Research and Implementation Center, Medical University of Silesia, Zabrze, POL; 4 Histopathology, Division of Dentistry, School of Medicine, Medical University of Silesia, Katowice, POL

**Keywords:** advanced peri-implantitis, biphasic calcium sulfate, bone defects, bone regeneration, vertical bone loss

## Abstract

The authors present the potential use of a hardening regenerative mineral cement in the treatment of bone defects complicated by peri-implantitis. The presented case concerns vertical alveolar ridge atrophy due to periapical inflammation of a tooth adjacent to an implant surface. Given the difficult clinical situation, the patient was consulted on the risk of regeneration, which was a priori associated with a high risk of failure. However, the use of a biphasic calcium sulfate cement, to the authors' surprise, resulted in a spectacular restorative effect. Although this is an isolated case, the authors believe that the biological properties of the cement significantly contributed to this success, which, according to the authors, would have been impossible with allograft or xenogenic bone in granular form and traditional guided bone regeneration techniques.

## Introduction

In a large number of implant procedures worldwide, a certain percentage of patients experience inflammatory changes in the bones and soft tissues around the implants, which can lead to their loss [1].

In the course of inflammatory processes in soft tissue and bone, broadly defined as peri-implantitis of various etiologies, the intensification of inflammatory cellular reactions, somewhat "assisted" by a bacterial component, also leads to a significant deterioration of clinical conditions due to loss of these tissues. A "gold standard" has not yet been achieved in the treatment of peri-implantitis, which means the long-term clinical effectiveness of "repair" procedures is not sufficiently predictable. Given the frequent need for radical removal of inflammatory lesions and infected implant surfaces, reinsertion of standard-length implants may prove impossible without prior reconstruction of the lost bone in these areas. This increases the patient's burden in terms of finances, time, and the potential failure of the reconstructive procedure. In line with the increasingly common principle of patient-centeredness, which aims to minimize surgical invasiveness, including in the oral cavity and jawbones, one alternative may be attempts at bone regeneration for small peri-implant defects. Again, there is no gold standard for this approach [2-4].

The authors would, therefore, like to present a case study using biphasic calcium sulfate mineral hardening cement as an interesting and highly effective alternative for the treatment of a mandibular bone defect, the significant atrophy of which resulted from inflammation of the tooth adjacent to an ultrashort Bicon® implant (Bicon Dental Implants, Boston, MA), complicated by advanced peri-implantitis. The mineral hardening cement Bond Apatite® (Augma™, Israel) has been used for augmentation [5].

## Case presentation

**Figure 1:**
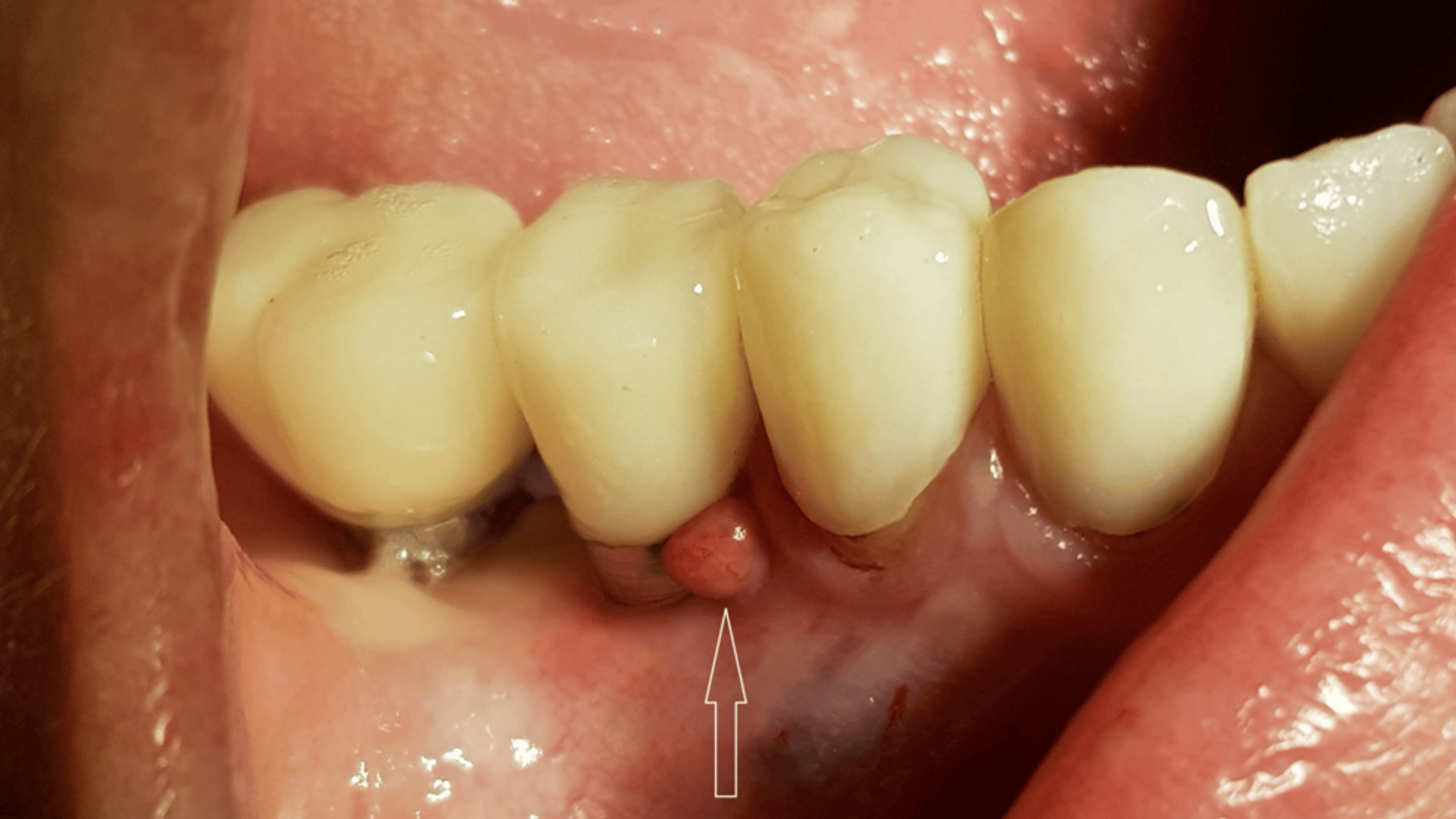
Clinical view of fistula in reg. 44-45

**Figure 2:**
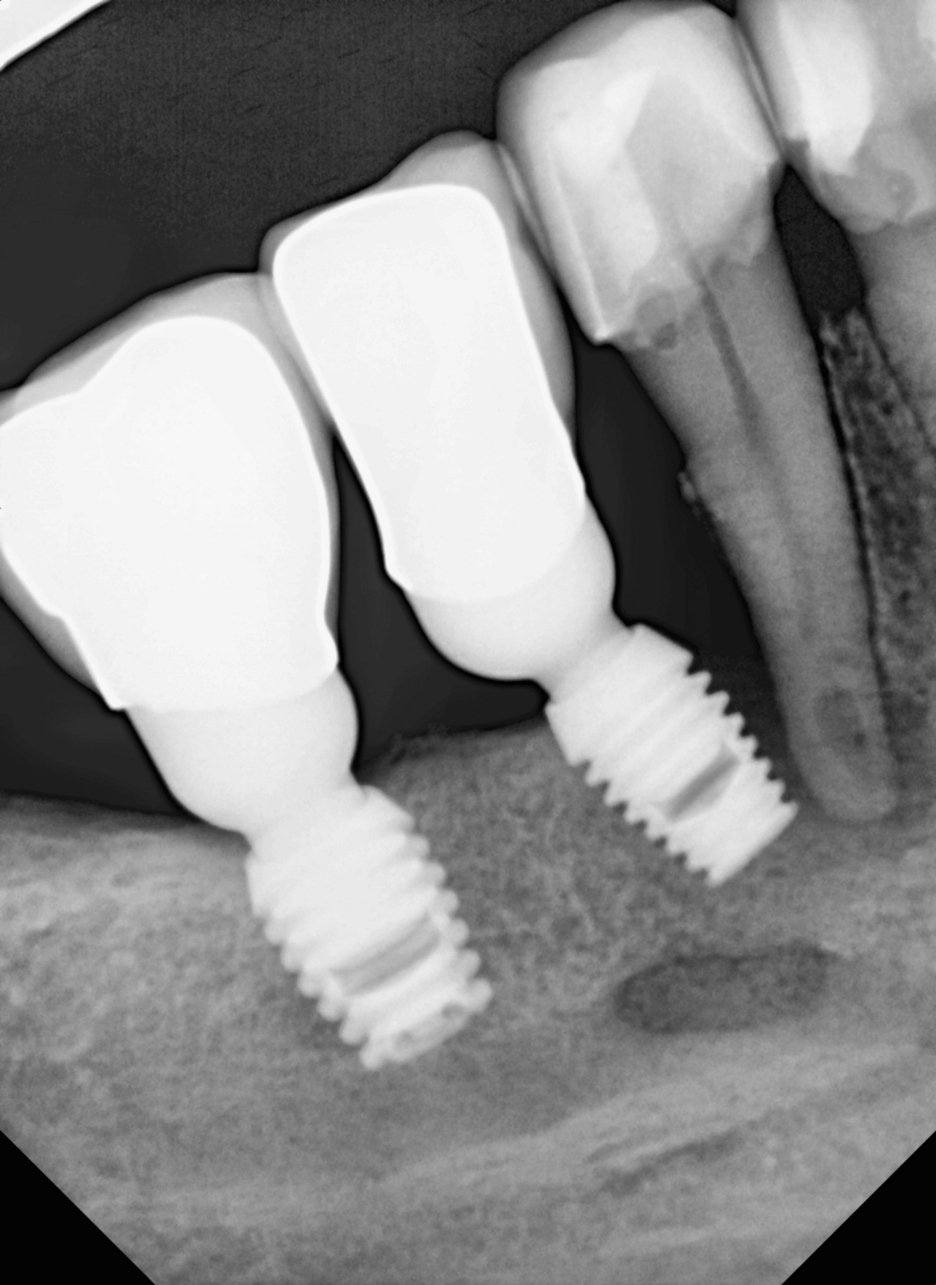
X-ray showing complete damage of septum 44/45

**Figure 3:**
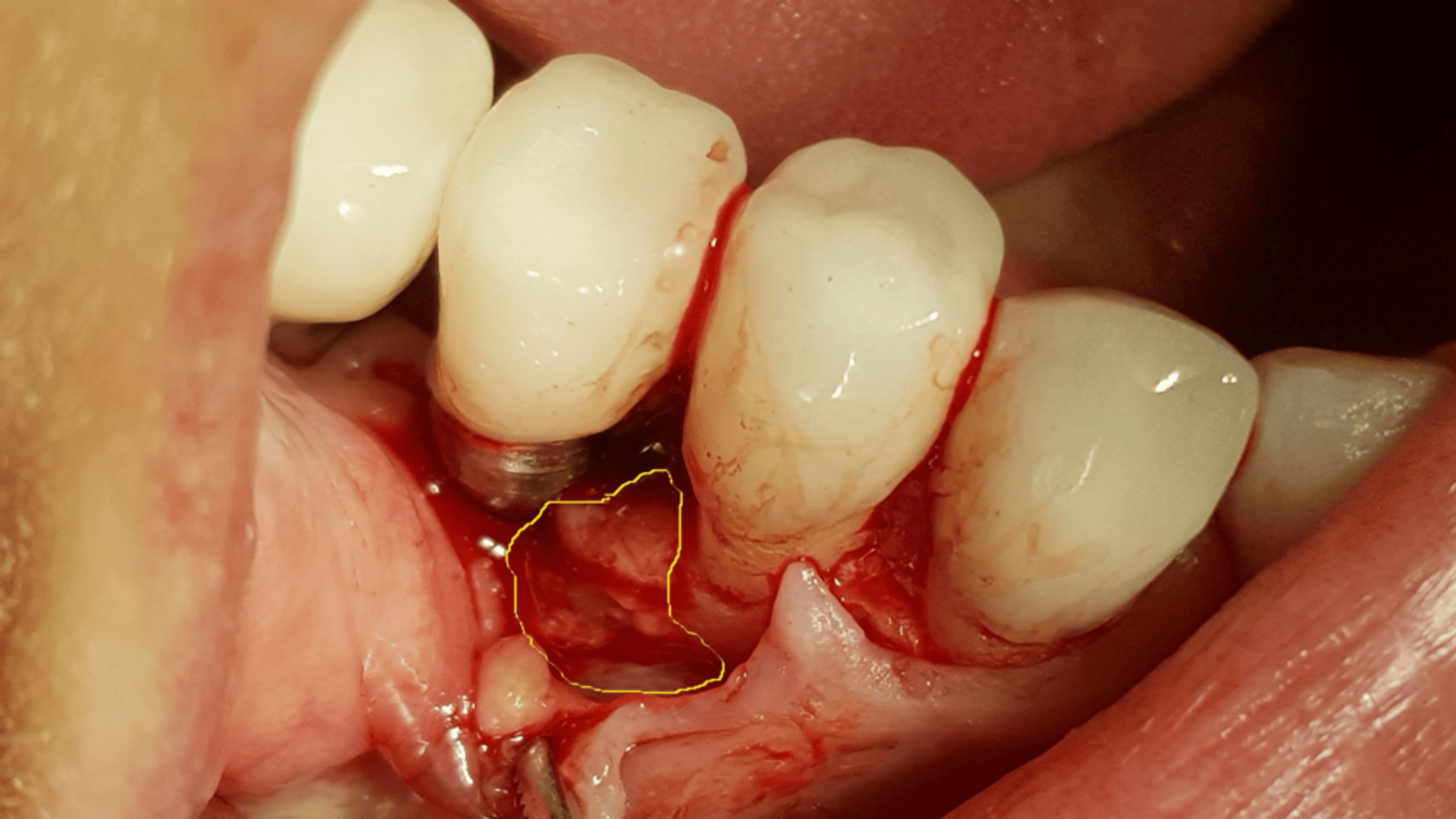
Big inflammation around tooth 44 and implant 45 is visible after flap opening

**Figure 4:**
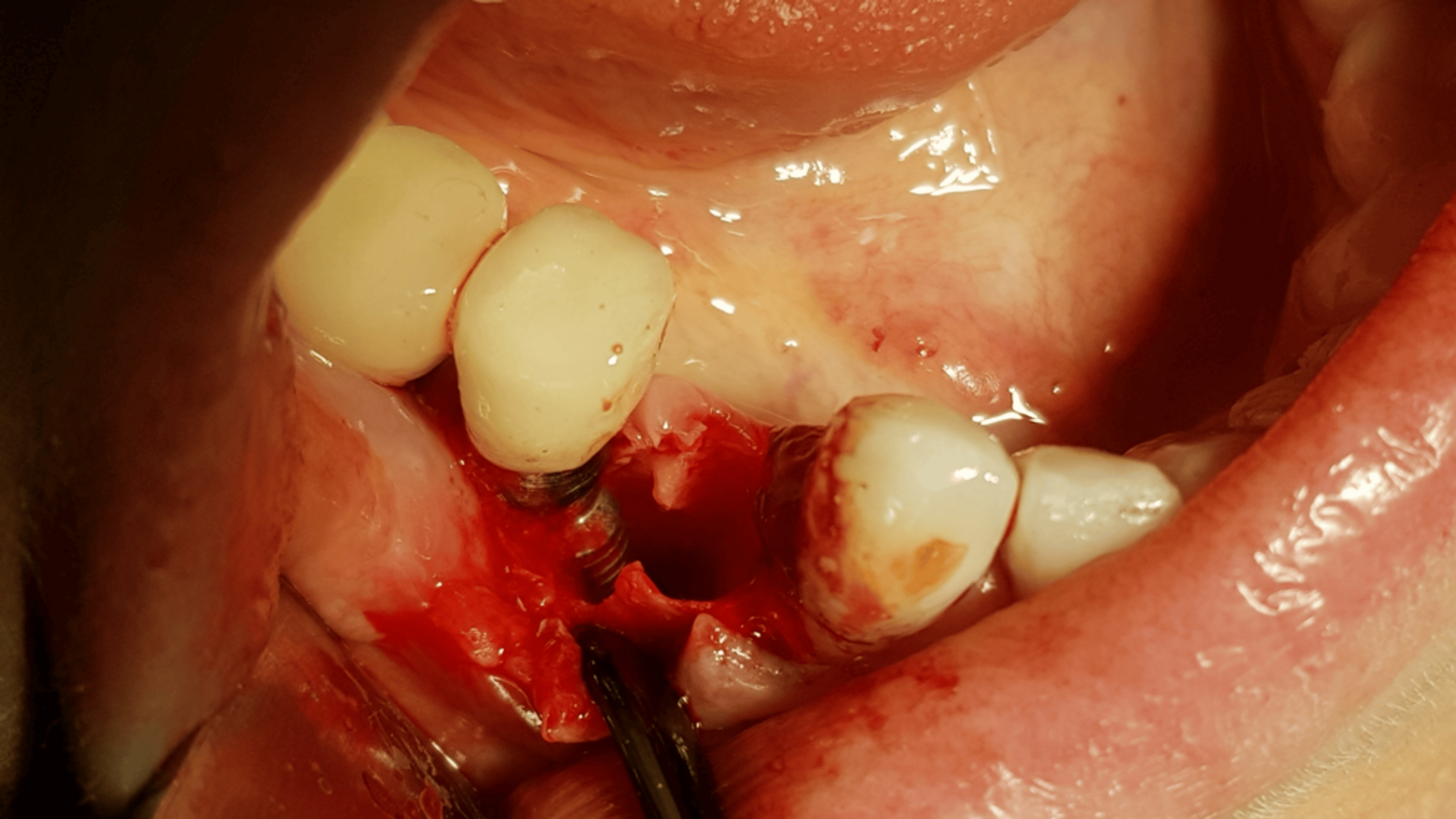
Bone defect showing totally exposed surface of the implant and damage of both cortical laminas

**Figure 5:**
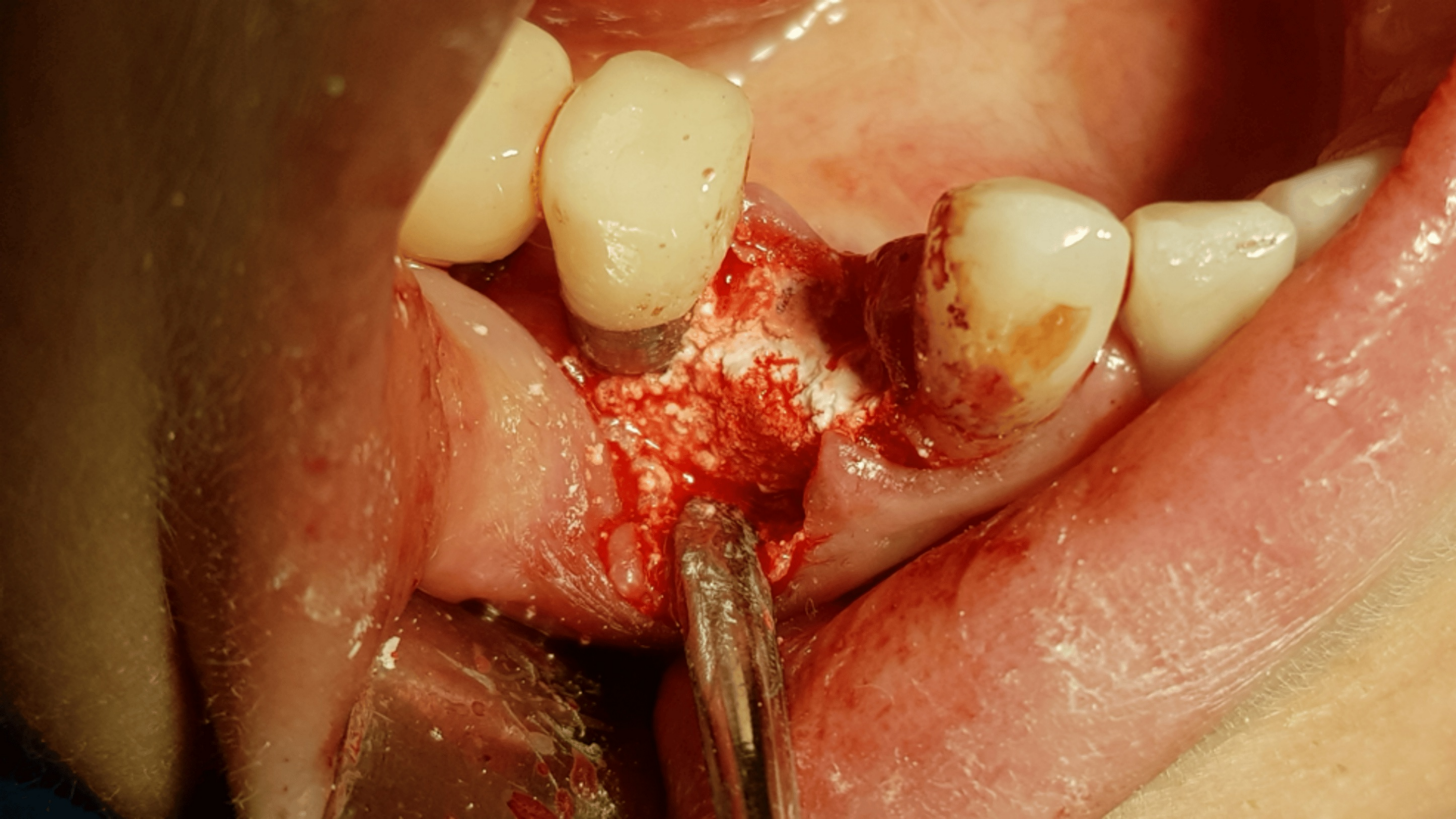
Bond apatite cement inside the defect

**Figure 6:**
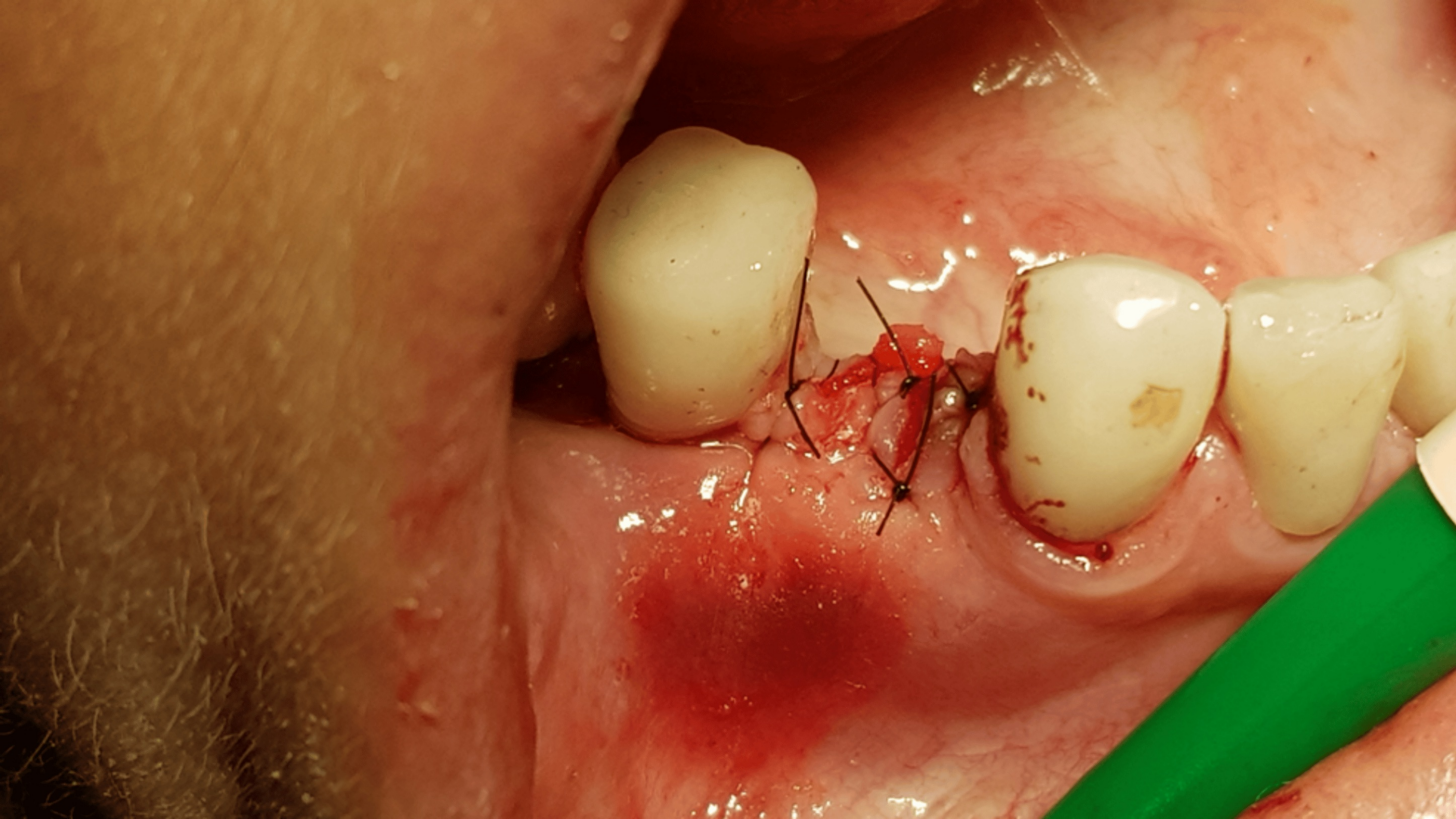
Suturation with only stretching of the mucosa

**Figure 7:**
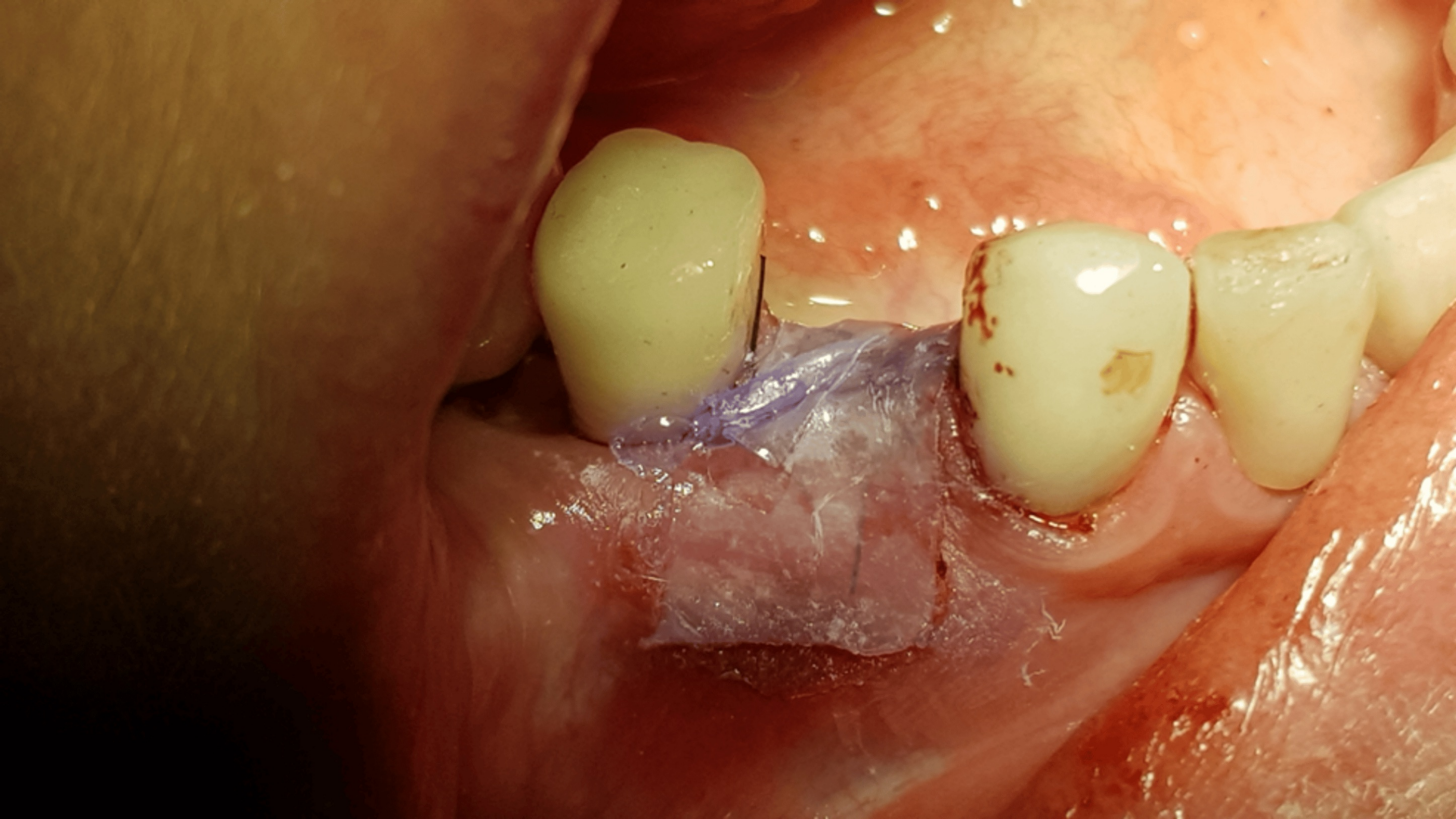
Augma-shield temporary dressing fixed with Periacryl 90 tissue glue

**Figure 8:**
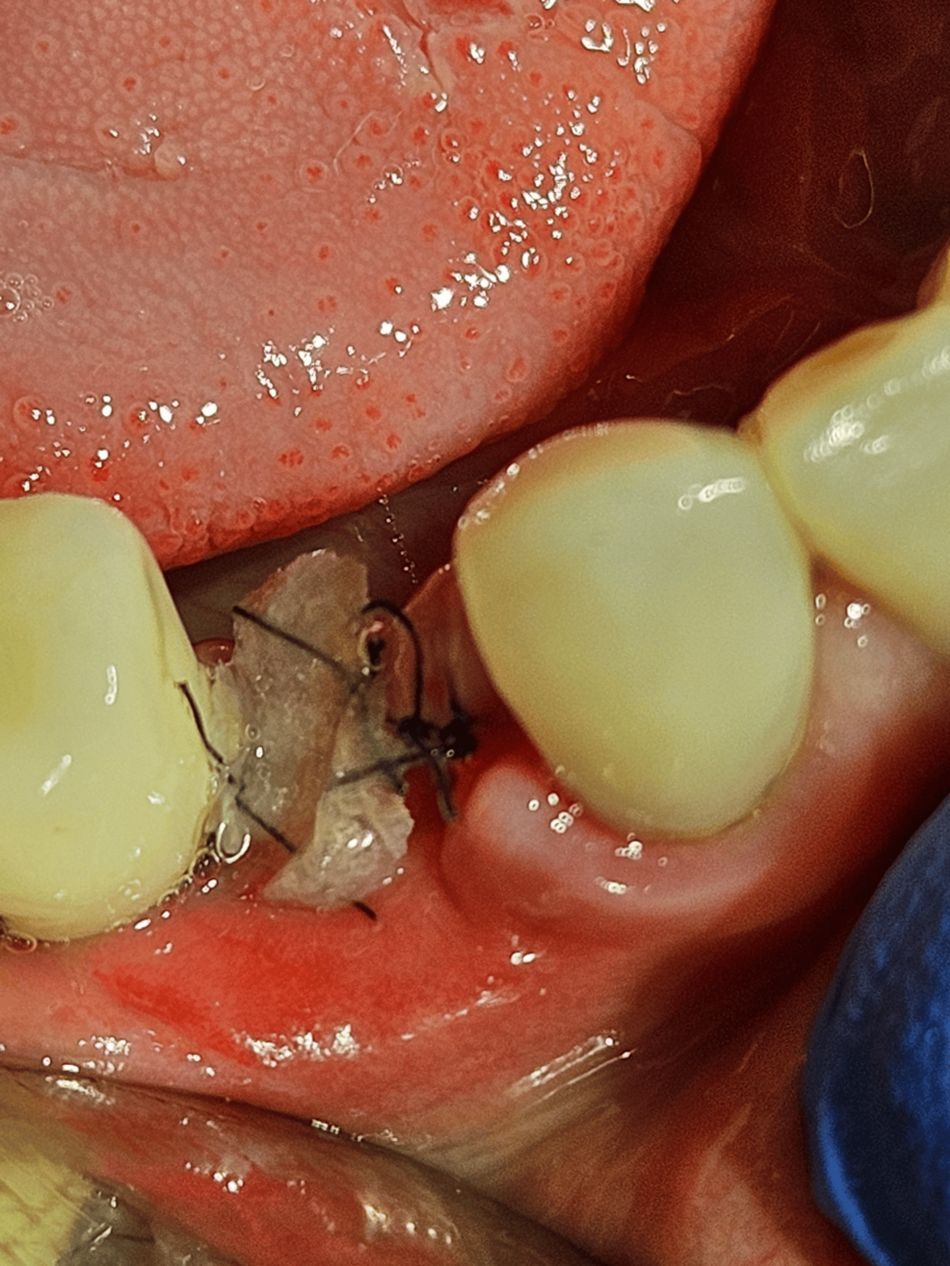
Mucosa seven days post-op

**Figure 9:**
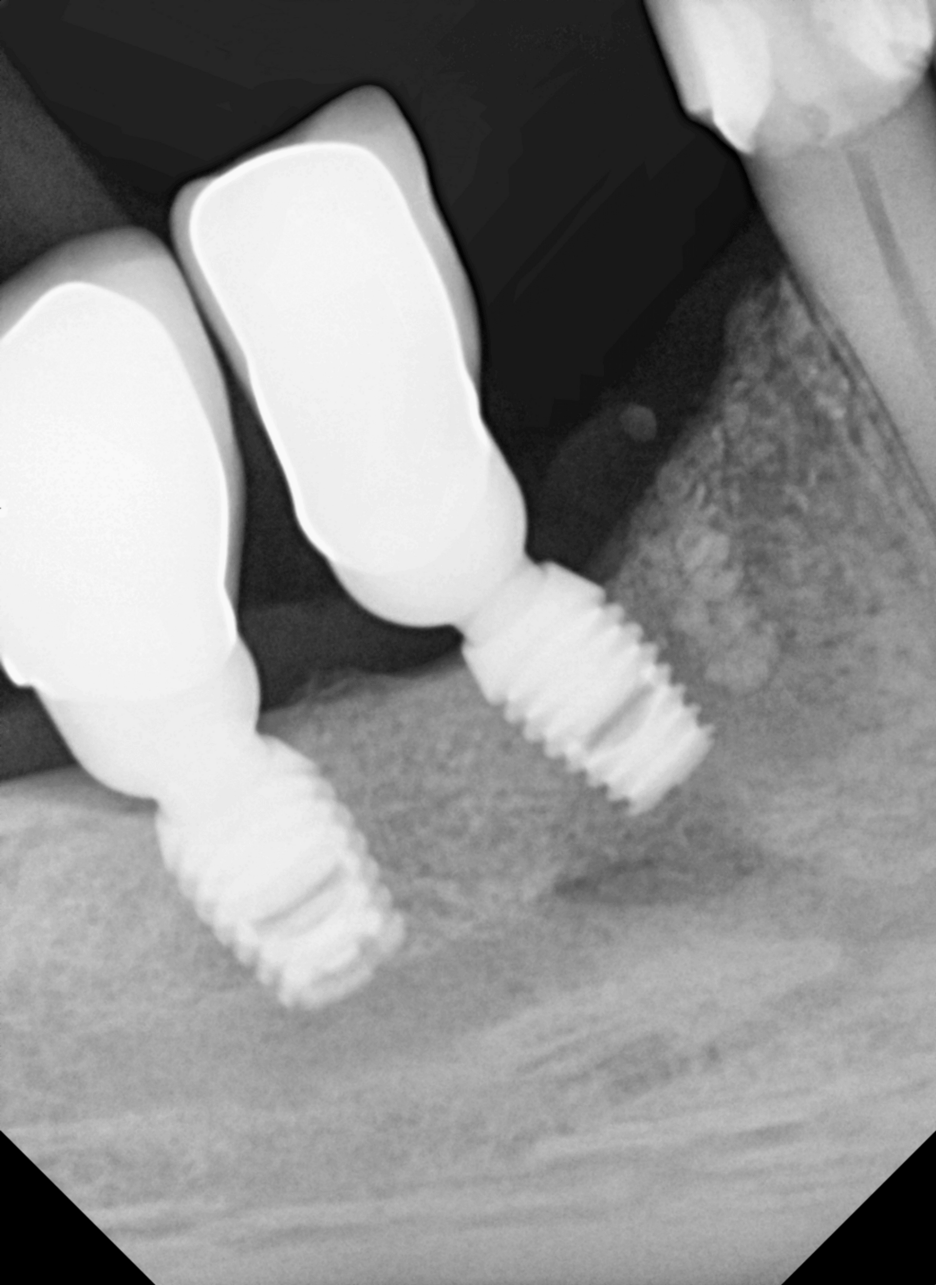
Control X-ray four months post-op

**Figure 10:**
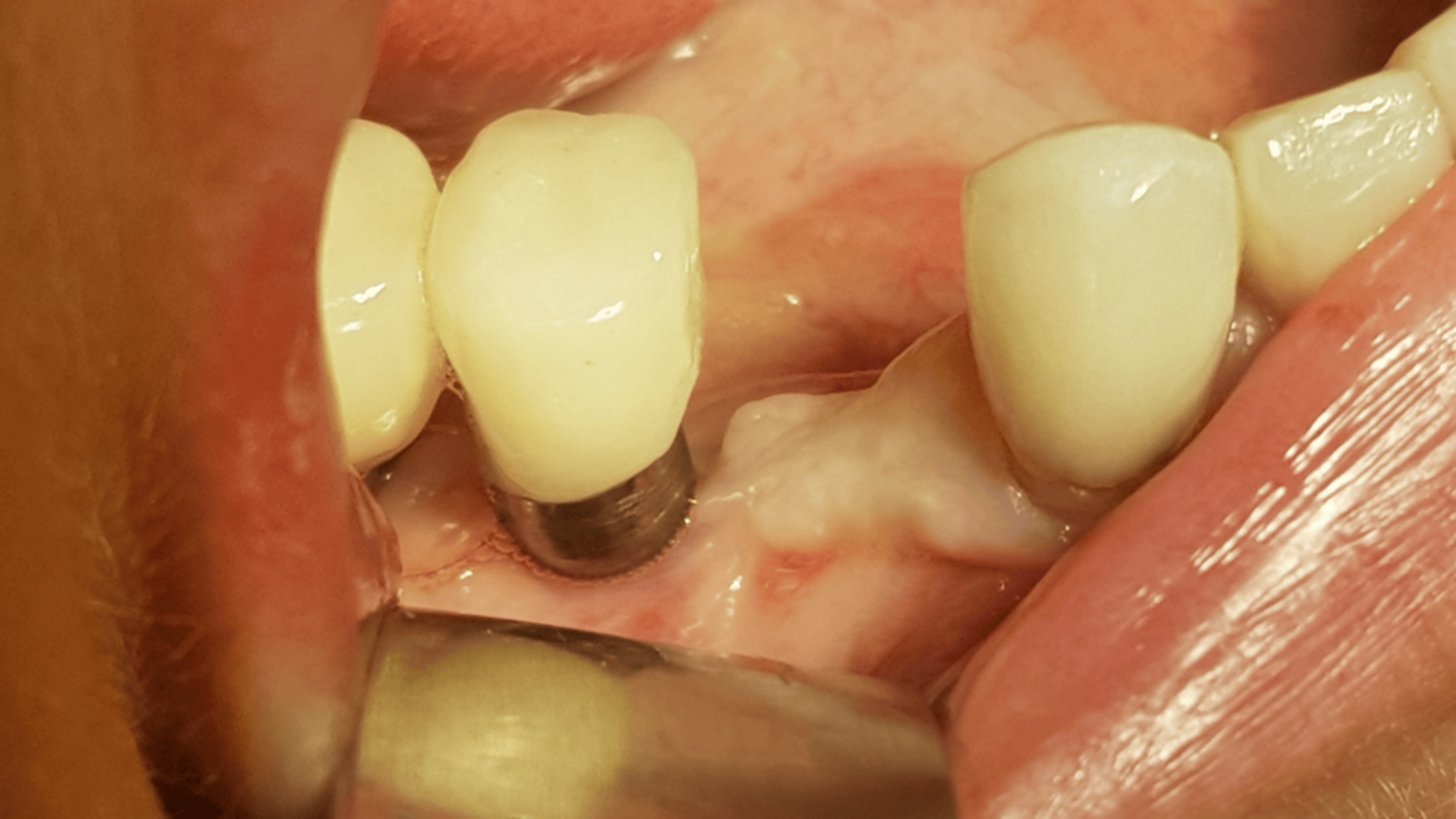
Mucosa four months post-op

**Figure 11:**
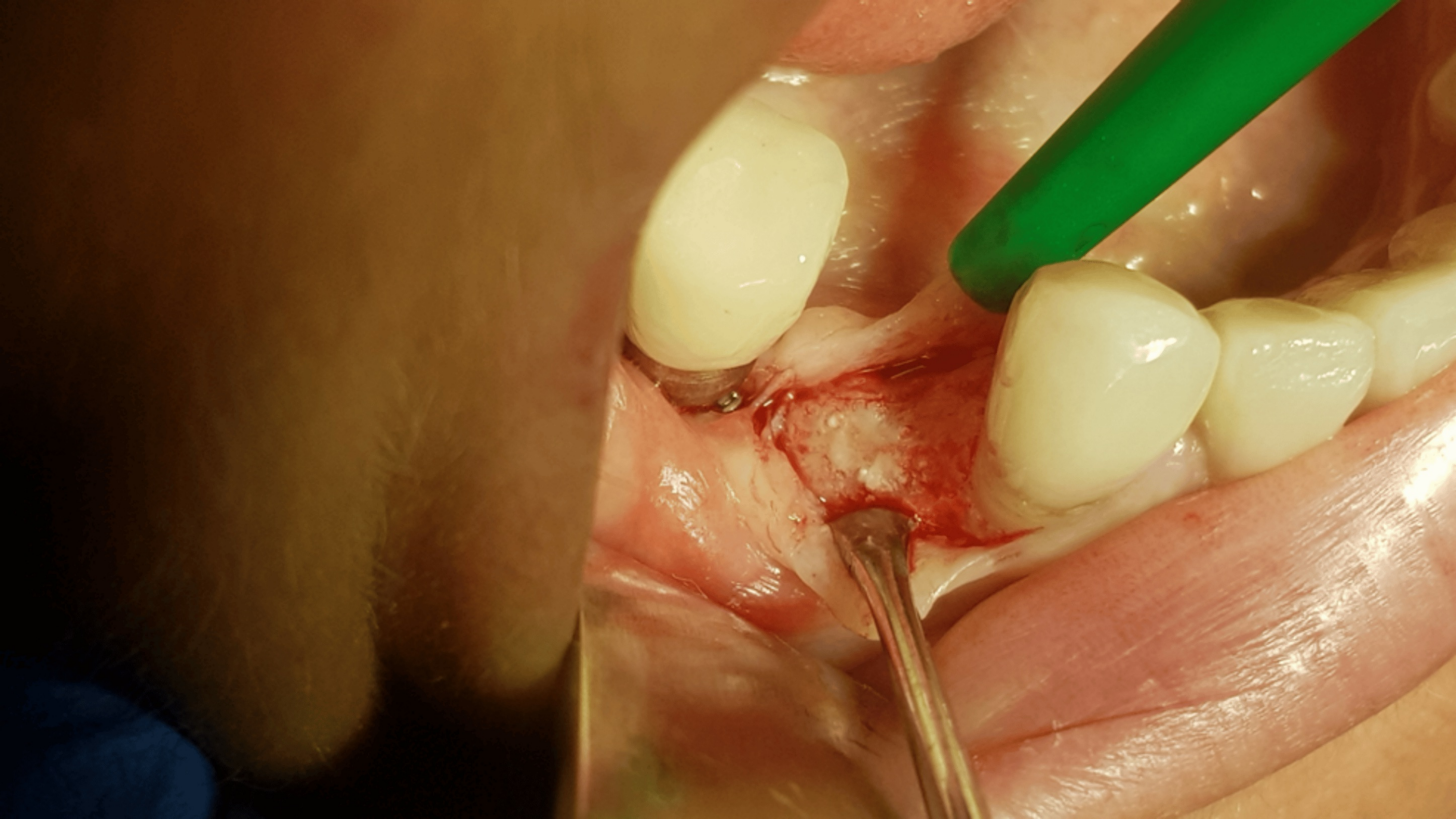
The complete regeneration of bone defect is visible after flap opening

A generally healthy 48-year-old female patient presented to our dental surgery clinic due to sudden pain in the right mandible, which had occurred several days earlier and was progressing. Clinical examination revealed pathological mobility of tooth 44 and a fistula around tooth 45 (Figure 1). An adjacent X-ray revealed advanced inflammatory changes around the apex of tooth 44 and in the interdental space extending to implant 45 (Figures 2, 3). Bone loss with destruction of both cortical plates exposed almost the entire mesial surface of the Bicon® implant (Bicon Dental Implants, Boston, MA). The extent of the loss was confirmed by cone beam computed tomography (CBCT; Ghent, South Korea) (Figure 4). Despite the high risk of failure, after considering the presented treatment options, the patient attempted to save the implant in the area of ​​45. The following treatment plan was established: extraction of tooth 44; debridement of the wound, with removal of inflammatory granulation tissue from the bone defect; disinfection and cleaning of the exposed surface of implant 45; and simultaneous augmentation of the defect. Depending on the outcome of these treatments, implantation of tooth 45 will be planned subsequently. All procedures were performed under local anesthesia with 4% Articaine with Noradrenaline (Septanest 1:100,000; 40 mg + 0.01 mg/mL, Septodont, France). In the first stage, tooth 44 was removed. Cortical damage was confirmed. After debridement and removal of inflammatory lesions from the bone and implant surface using titanium instruments, the exposed implant fragment was disinfected with clindamycin solution and left in the defect for five minutes (clindamycin 600 mg/mL, MIP-Pharma, Poland). The defect was then filled with regenerative cement (1 cm^3^; Bond Apatite, Augmabio, Caesarea, Israel) (Figure 5). After stretching the mucosa, the wound edges were brought approximately 3 mm closer together and sutured (4-0 polydioxanone, round needle, 16 mm length, ½ circle profile) and additionally covered with an Augma-Shield dressing, which was adhered to the wound using tissue glue Periacryl 90 (GluStitch Inc., Delta, Canada; Figures 6, 7). After seven days, soft-tissue granulation was achieved (Figures 8-10). A six-month healing protocol was adopted. After this period, clinical and radiological follow-up completely surprised both the patient and the surgeon. The results were astonishing: complete regeneration of the defect, including both cortical plates and the area of ​​the exposed surface of the Bicon® implant. Therefore, planned implantation was performed. Intraoperatively, full regeneration of the damaged cortical plates was confirmed, along with an optimal bone ridge width of approximately 6 mm, allowing for the creation of a bed for a standard screw implant with a diameter of 3.75 mm and a length of 10 mm with primary stabilization of about 25 Ncm (Figure 11).

**Figure 12:**
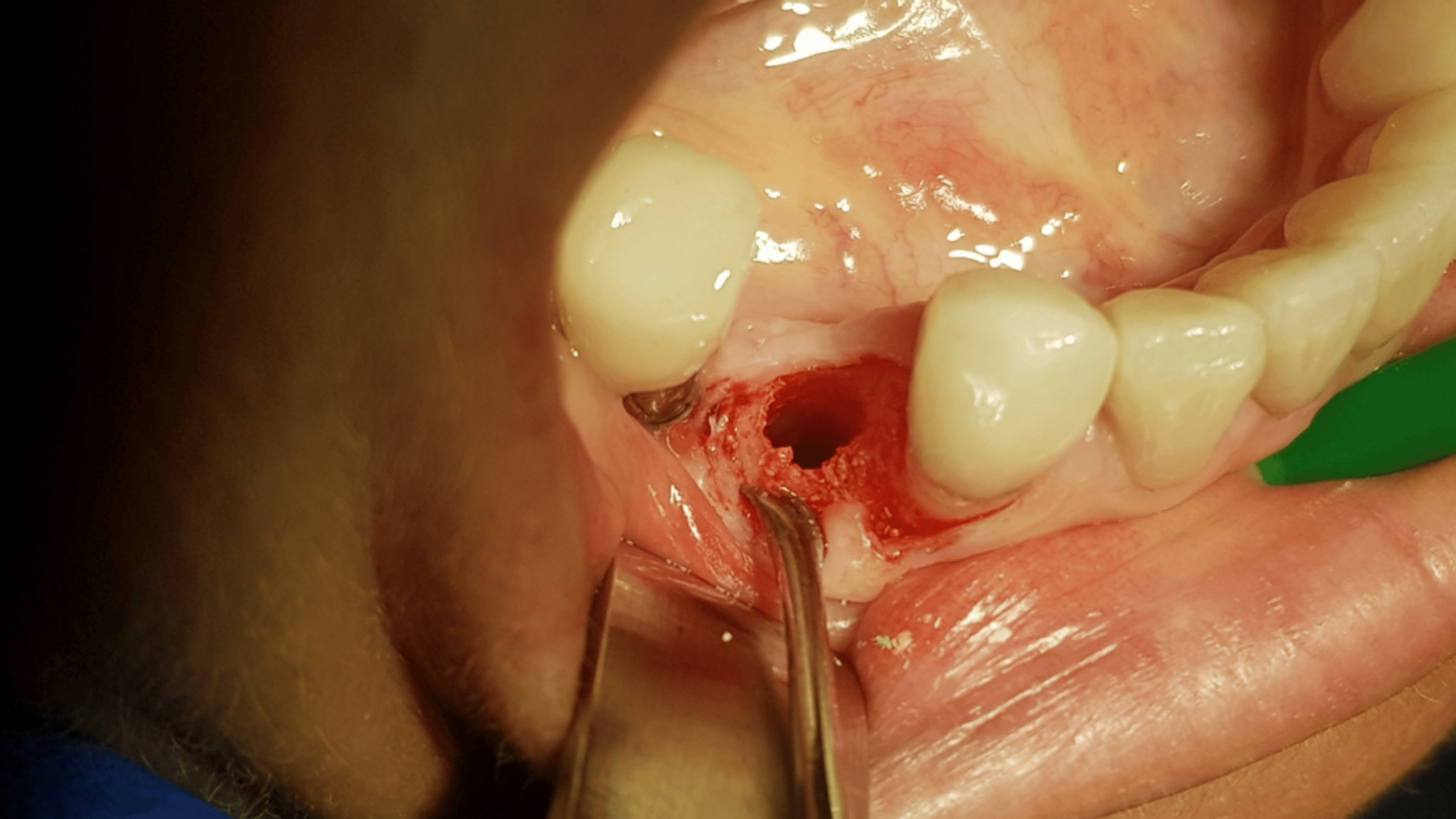
Preparing the implant hole (the bone is bleeding)

**Figure 13:**
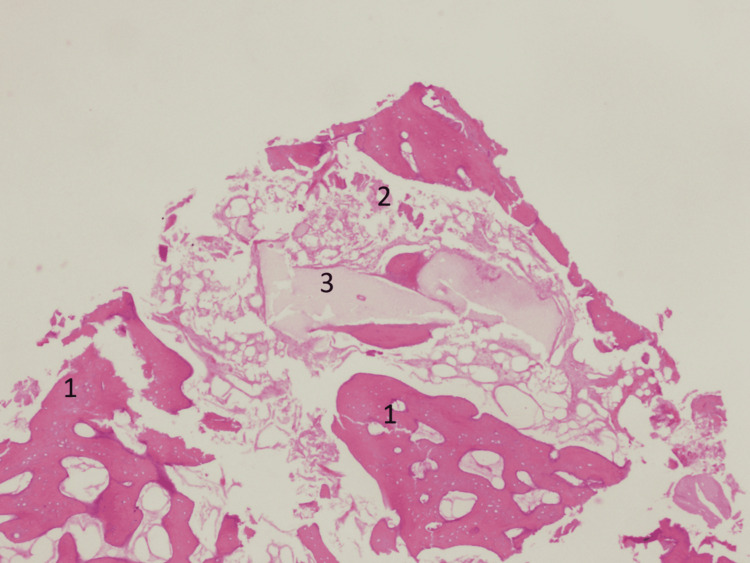
Microphotography showing good rebuilding of the bone. Hematoxylin & eosin steaming, focus 100×. 1: new bone. 2: bone marrow. 3: hydroxyapatite crystals

**Figure 14:**
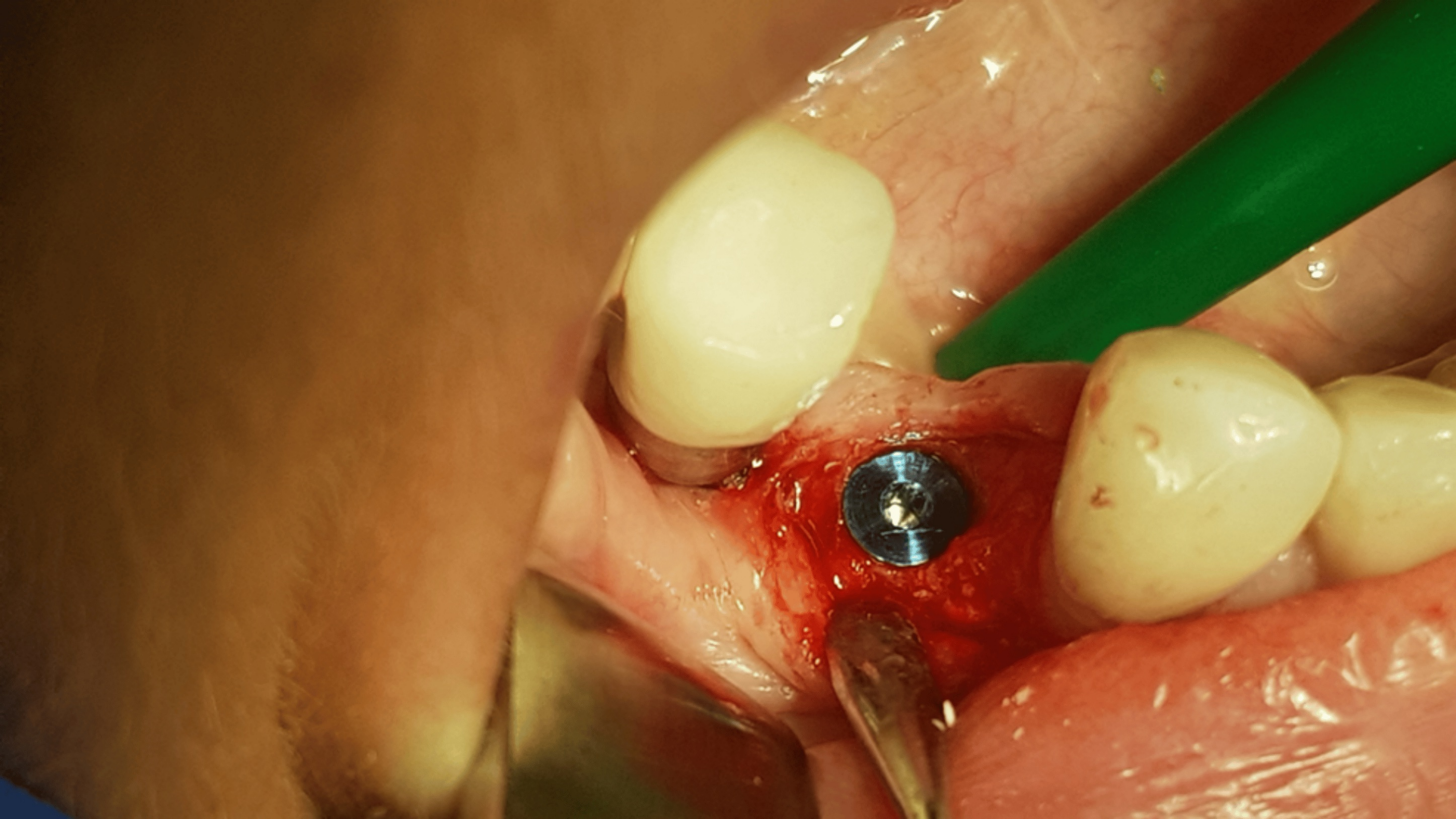
Implant inside with cover screw (Noris Tuff: D, 3.75; L, 10 mm)

**Figure 15:**
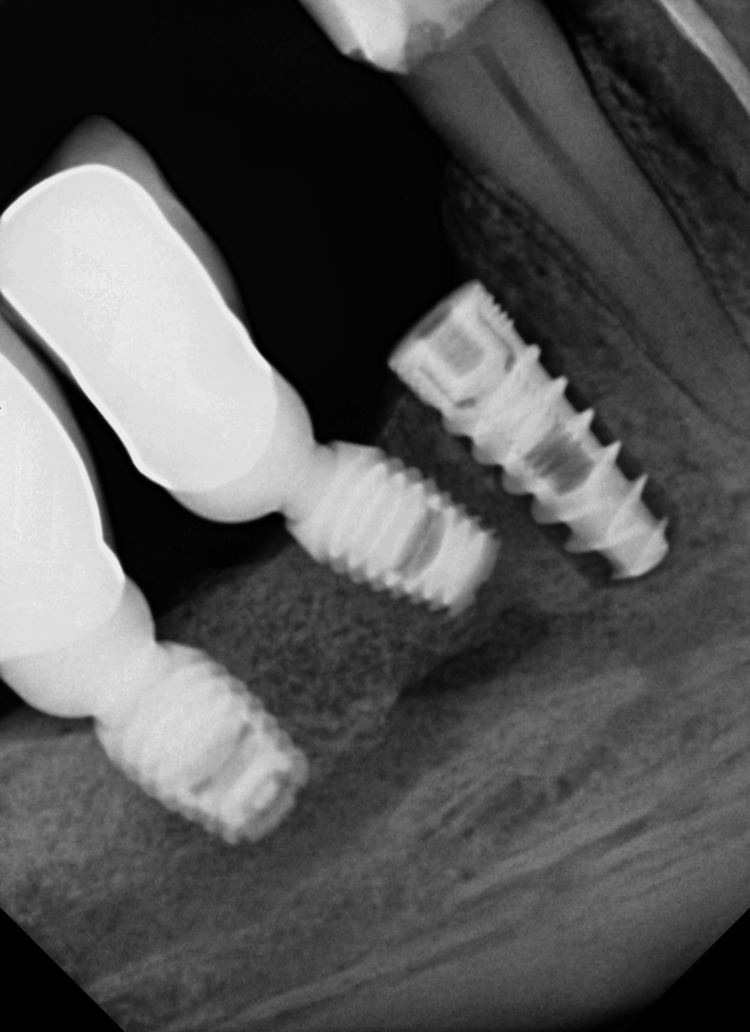
Control X-ray postinsertion implant (D: 3.75 mm, L: 10 mm)

**Figure 16:**
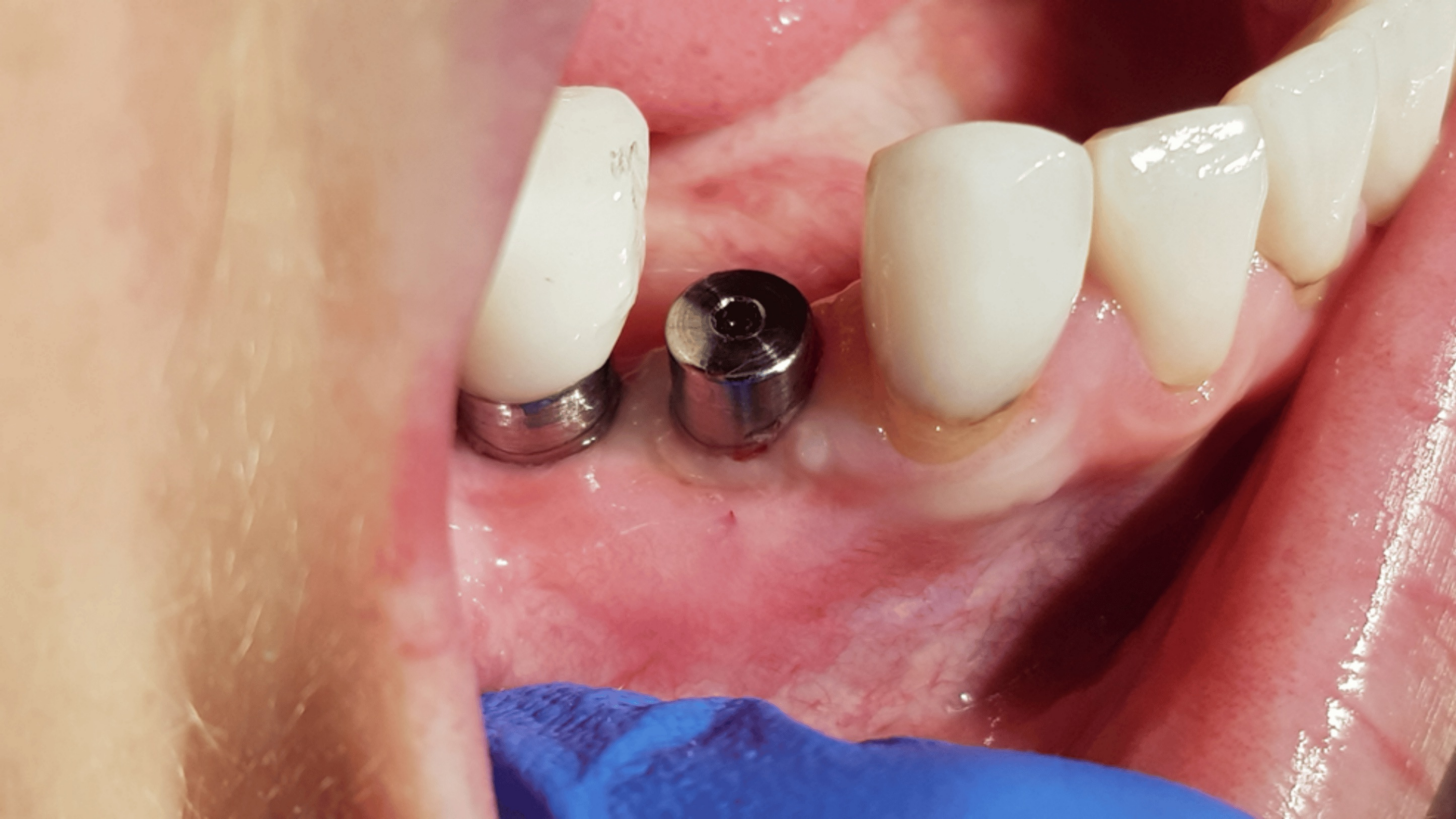
Healing screw visible in the mucosa in the next three months seven days after opening

**Figure 17:**
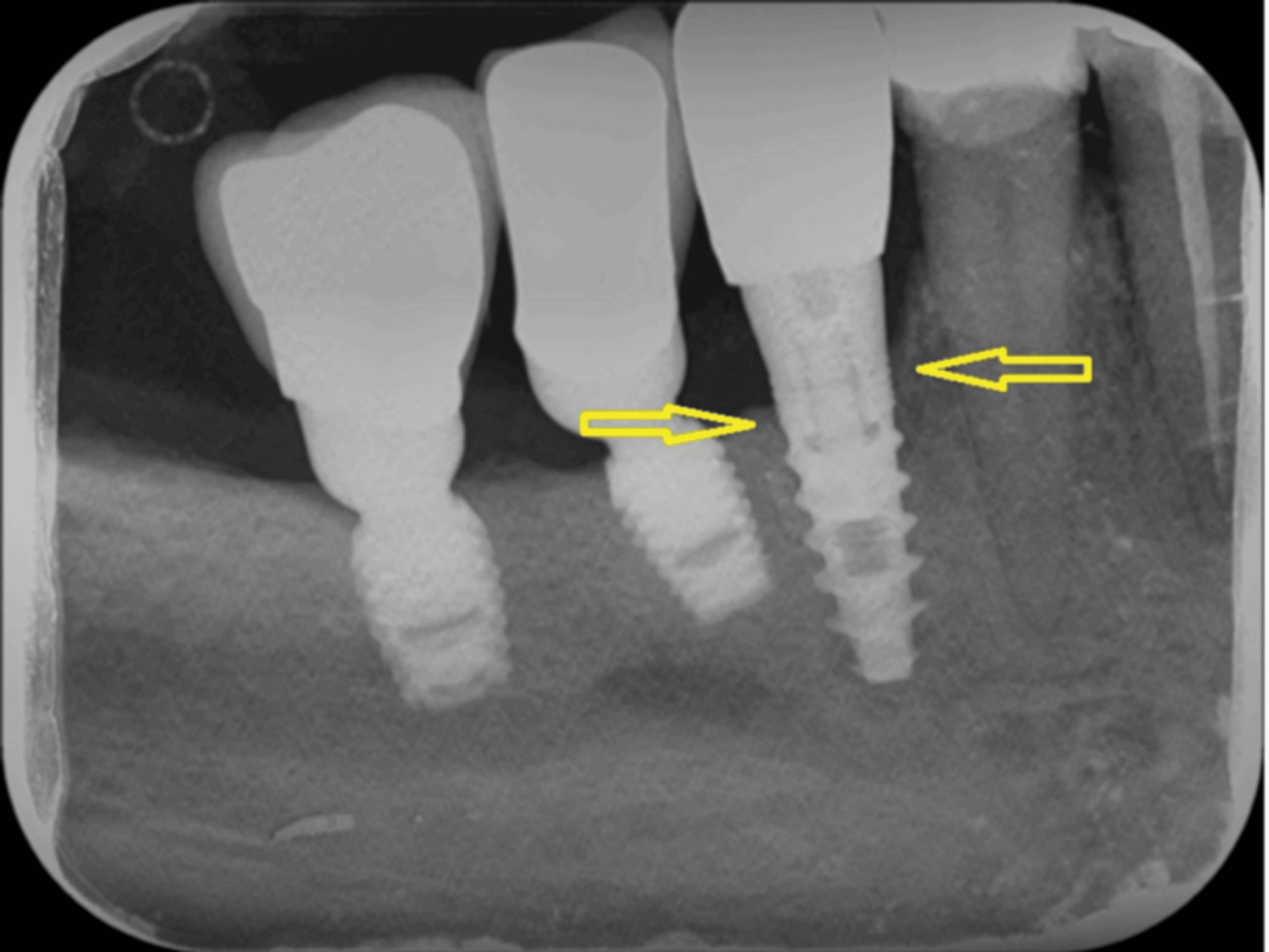
Control X-ray three months after prosthetic restoration. The new bone is rebuilt and stable

**Figure 18:**
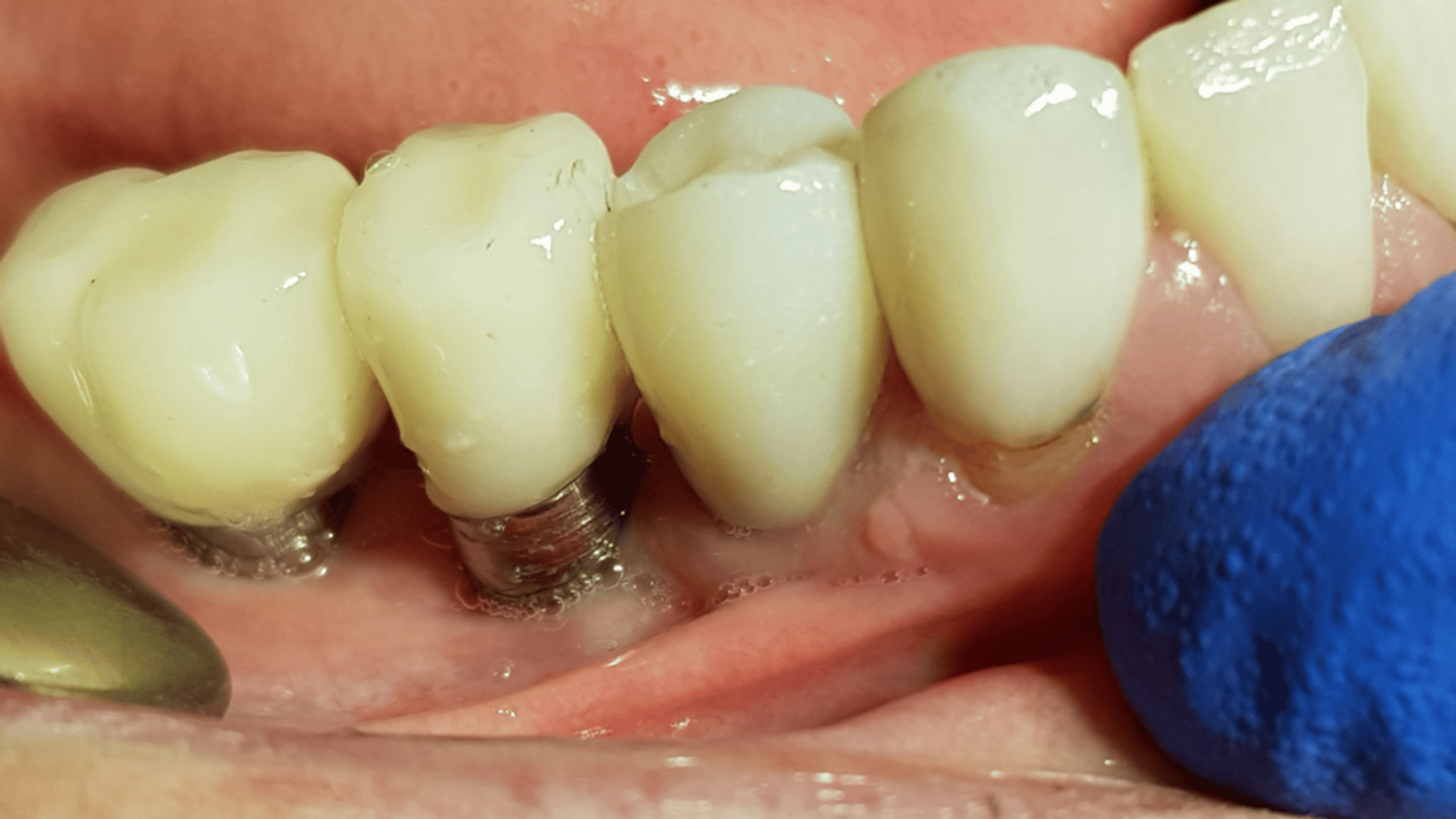
Mucosa three months after prosthetic restoration

The implant was inserted into the bone with a force of approximately 20 Ncm. During bed preparation, the bone tissue bled profusely, which may also indicate good revascularization of the regenerated defect (Figures 12, 13). A bone sample was also collected for histological evaluation. The wound was sutured (polytetrafluoroethylene 4-0, reverse cutting needle, 3/8 circle profile, length 16 mm). Osseointegration of the 45 implant is progressing well, and a prosthetic suprastructure will be installed as planned. Furthermore, no secondary inflammatory complications were noted. The implant has been opened and equipped with a cover screw (Figure 14). Clinical and X-ray control after three months confirmed the proper functioning of the implant and prosthetic superstructure (Figures 15-18).

## Discussion

In oral implantology procedures performed, a significant percentage of cases involve inflammatory complications of the bone and soft tissue around implants, broadly defined as peri-implantitis. No gold standard has yet been identified in regenerative treatment, leaving surgeons struggling with failures and experimenting with augmentation, with varying degrees of success and clinical durability. Therefore, mineral and hardening bone cements, including Bond Apatite®, used in the above case, can be an effective alternative to autologous or allogeneic augmentation. Furthermore, the material can be highly effective in cases such as the one presented above, where bone loss prevented standard immediate implant placement and posed a threat to the support of the adjacent implant. The use of hydroxyapatite mixed with biphasic calcium sulfate allowed for the reconstruction of the bone defect and salvage of the inflamed implant. The authors believe this was possible due to the material's hardening consistency, osteoconductive properties, and high bacterial resistance. Although this is an isolated case, it is possible to use this cement in similar situations.

It is known that a mixture of biphasic calcium sulfate and hydroxyapatite has been known and used for decades, starting in the 19th century. However, they contain one of the forms of calcium sulfate: dihydrate or hemihydrate (α or β form). The formula of the regenerative cement used here is unique because it contains both forms of calcium sulfate: dihydrate and hemihydrate, which allows for optimal binding and modeling in bone defects in the presence of blood, saliva, proteins, etc. The name "biphasic calcium sulfate" was patented in 2010. Moreover, that mineral cement is made using advanced technology and is protected by a patent pending. The procedure for preparing and treating the recipient site eliminates the need for barrier membranes and therefore does not impair periosteal nutrition of the newly forming tissue. However, the material itself provides a mechanical barrier to the ingrowth of reparative connective tissue into bone defects, significantly reducing the risk of secondary inflammatory complications. The bacteriostatic properties of biphasic calcium sulfate are also extremely important, especially when used in a contaminated environment such as the oral cavity. This reduces the risk of secondary infection, even with partially exposed wounds. This also explains why the manufacturer recommends that primary, tight wound closure is not absolutely necessary. The authors' several years of experience in bone defect regeneration are presented, for example, in a study evaluating the six-month follow-up of single-stage treatment of radicular cysts in 30 patients, including filling the bone defects with Bond Apatite® regenerative cement. In the control group, 30 patients underwent similar procedures using other biomaterials. A three-point scale was adopted to assess postoperative wound healing, with the following scores after seven days: 0° indicated a tight wound, I° indicated dehiscence up to 10 mm (from one wound edge to the other) and the wound left to heal by granulation with local aseptic support, II° indicated wound dehiscence of more than 10 mm, requiring repeated surgical intervention and general pharmacology.

Comparative analysis revealed one I° failure in the study group on day 5, while in the control group, four I° cases and another two II° cases were observed within five days. In the remaining patients, a six-month follow-up revealed no secondary inflammatory complications, and radiographic remodeling and new bone growth in the defects were normal [6].

The authors' experience is also consistent with the results of clinical studies and studies conducted by other teams using regenerative cements containing two-phase calcium sulfate, as briefly discussed below.

Some studies available in the literature confirm the regenerative capabilities of the presented bone cement and its wide range of applications. A good example is the work by Yahav et al. The study examined clinical, radiological, and histological observations of bone tissue after using this biomaterial in the regeneration of various defects in the jawbone. This extensive study demonstrated excellent results in the regeneration of bone defects following tooth extraction, periodontal defects, and vertical and lateral augmentation (ridge reconstruction, sinus lift, and treatment of odontogenic cysts). Importantly, histological analyses of selected augmentation procedures demonstrated biomaterial resorption and new bone formation, with an average time of three months after the procedures [7].

New, inspiring reports are also emerging regarding the regenerative potential of thin alveolar ridges. A clinical study by Ferreira and Kurtzman demonstrates the high clinical efficacy of Bond Apatite® in restoring lost ridge deficits in 14 edentulous patients combined with immediate implant placement in the maxilla or mandible. Clinical and radiographic follow-ups were performed every six months. The authors achieved acceptable regenerative results and survival of all implants without inflammatory changes in the bone or soft tissues within one to five years of augmentation [8].

In addition, the study by Baranes and Kurtzman recommends the hardening regenerative cement for various clinical applications, including the regeneration of alveolar process defects, where the complete absence of a vestibular wall forces clinicians to augment these defects as the first step in preimplantation procedures. In this study, defect regeneration was achieved, allowing for implant placement at an average of four months [9].

Comparable clinical results in the augmentation of large bone defects in the lateral maxilla using biphasic calcium sulfate (Surgiplaster, Classimplant®, Rome, Italy) were reported by Laino et al. Lateral sinus lift procedures were performed in 25 patients. A significant increase and remodeling of bone tissue by an average of 8.21 mm was observed during the six-month follow-up period. The authors consider the tested biomaterial safer than, for example, autogenous bone and prefer it for this type of procedure [10].

Once again, Baranes and Kurtzman presented an innovative technique for the regeneration of the maxillary alveolar process by mobilizing the sinus mucosa via a crestal approach. Bond Apatite® mineral cement and original instruments were used in 51 cases. The initial alveolar bone height averaged 1.5 mm. After a four-month period, bone regeneration was confirmed radiologically on CBCT and clinically during implant placement, ranging from 4.5 to 7 mm in height, which, with an appropriate ridge width, allows for implant placement [11].

In the discussion of bacterial resistance, it is also worth mentioning a remarkably interesting experimental study by Mistry et al., conducted in an animal model. It involved the use of a regenerative cement containing, among other things, calcium sulfate and biphasic calcium phosphate as an antibiotic carrier. Chronic inflammation of the tibia bone was induced by methicillin-resistant *Staphylococcus aureus* in 42 rabbits. The above cement and a polymethyl methacrylate (PMMA) material were then introduced into the inflamed areas. Observations were conducted for 90 days. The authors obtained very interesting results. They noted significantly better drug release over time from the composite cement and its faster biodegradation compared with PMMA. Although cement containing calcium sulfate and phosphate has, according to the researchers, lower mechanical strength, high antibiotic concentrations were observed around bacteria at day 42, and significantly higher concentrations where composite cement was applied. It was hypothesized that cement containing calcium sulfate could be successfully used to support the treatment of osteomyelitis and prevent infection following osteosynthesis of fractures. This, of course, requires expanding the research panel in both directions [12].

To conclude, Lombardo et al.'s report presents exceptionally interesting results on peri-implantitis treatment. A three-year follow-up study of 15 patients treated for inflammatory symptoms and bone loss around 17 implants was conducted. The average bone loss was 3.04 mm, but the use of a formula containing calcium sulfate, among other ingredients, reduced bone loss by an average of 1.74 mm. Radiological assessments also revealed an average increase in new tissue of 55%. Although only a histological examination could clearly confirm new bone growth in contact with the implant surface, the team believes that using a biomaterial without barrier membranes simplifies and shortens the entire treatment process [13].

## Conclusions

To summarize the brief considerations above, it should be emphasized that, based on the presented case, the literature, and the authors' own experience, the use of Bond Apatite® mineral cement effectively supports bone regeneration procedures, including the reconstruction of cancellous bone and cortical lamina in periapical inflammation complicated by peri-implantitis. In the presented case, an optimal regenerative effect was achieved, enabling planned implantation using a standard intraosseous implant. However, it is important that the use of Bond Apatite® regenerative cement, as in the presented case and based on scientific and clinical reports, demonstrates promising outcomes and high effectiveness in regenerating various jawbone defects. Additionally, its significant advantages include a simple application protocol, a minimally invasive surgical technique for preparing the recipient site, and the material's bacterial resistance. Economic considerations are also important for patients. In the authors' opinion, further long-term clinical studies, increasingly broader observations of our own cases, and the already available favorable results of histological analyses will allow us to take up the challenge and introduce a new alternative in guided bone regeneration.
